# Mechanisms of Aβ induced synaptic toxicity

**DOI:** 10.1186/1750-1326-8-S1-O26

**Published:** 2013-09-13

**Authors:** Edward H Koo

**Affiliations:** 1 Department of Neurosciences, University of California, San Diego, La Jolla, CA 92093, USA

## 

Increasing evidence favors the synapse as one of the initial sites of neuronal damage by amyloid β-protein (Aβ) and such synaptic damage is thought to underlie the cognitive deficits seen in Alzheimer’s disease. A decade ago, Roberto Malinow and colleagues published a seminal paper proposing that neuronal activity augments processing of the amyloid precursor protein (APP) by β- and γ-secretases to enhance Aβ production and release. In turn, Aβ then depresses synaptic activity. Thus, an interesting positive feed forward and negative feedback loop is created during periods of neuronal activation. Since publication of this report, a number of elements within this intriguing pathway have been confirmed by other laboratories in both the *in vitro* and *in vivo* settings. Specifically, there is compelling evidence that neuronal activity is associated with enhanced Aβ generation and subsequent amyloid deposition. Similarly, Aβ has been shown by multiple investigators to depress synaptic activity and synaptic plasticity. In this presentation, I will summarize some recent work examining the mechanisms that underlie both pathways. In particular, among the many proposed pathways of Aβ toxicity, I will discuss the potential involvement of APP in Aβ-initiated synaptic damage.

